# Does mosquito mortality in WHO insecticide susceptibility tests relate to mosquito mortality in LLIN experimental hut studies?

**DOI:** 10.1186/1475-2875-11-S1-O43

**Published:** 2012-10-15

**Authors:** Olivier JT Briët, Nakul Chitnis, Melissa Penny

**Affiliations:** 1 Department of Epidemiology and Public Health, Swiss Tropical and Public Health Institute, Basel, Switzerland; 2 University of Basel, Basel, Switzerland

## Background

With the rise of insecticide resistance, there are concerns that insecticide treated nets (LLINs) will become less effective against malaria transmission. The WHO insecticide susceptibility (tube/ cylinder) test is a standard for estimating mosquito susceptibility to insecticide. The test measures the proportion of mosquitoes that die when exposed to an insecticide with a certain concentration. Whereas, with fully susceptible mosquito populations, mortality is 100% with 0.05% deltamethrin, but with resistant populations, mortality can be below 15%. However, little is known how susceptibility results in these tests relate to effectiveness of LLINs in the field. Experimental huts provide semi-field conditions and provide a more direct way of estimating the effect of susceptibility of mosquitoes to interventions than WHO susceptibility tests. This work assesses the degree to which mortality in WHO susceptibility tests correlates to mortality in experimental hut studies with LLINs.

## Materials and methods

For seven wild mosquito populations, published experimental hut results [1-5] were available for untreated, twenty times washed, and unwashed nets, for which the insecticide content was also measured. Nets were either artificially holed (hut assays for six populations), or intact (hut essays for one population). For six of these populations, mortality in WHO susceptibility tests were also published. These data were used to estimate deterrence (reduction in hut entry), the proportion of mosquitoes attacking of those entered, and pre- and post prandial mortality, as a function of LLIN insecticide concentration and as a function of holed area. These variables were also used to define personal protection (reduction in bites received), and corrected mortality (mosquito mortality), as a function of insecticide concentration and holed area. Using these parameterizations, the corrected mortality in experimental huts for all populations was calculated for a standard net with a fixed deltamethrin concentration of 17.44 mg.m^–2^ and 96 cm^2^ holed area.

## Results

The Pearson correlation coefficient for the correlation between corrected mortality values and mortality in WHO susceptibility tests was strong and positive at r=0.92. However, for personal protection, the correlation was much weaker (r=0.54).

## Conclusions

Our analysis concludes that the WHO susceptibility tests give a good indication of the killing efficacy of LLINs in semi-field conditions. However, the efficacy of LLINs in the field is not only determined by direct mortality, but also by personal protection and deterrence, for which correlations are much weaker.
